# Annual reviews: Recent advances in analytical sciences

**DOI:** 10.1002/ansa.202200011

**Published:** 2022-04-15

**Authors:** Sebastiaan Eeltink

**Affiliations:** ^1^ Department of Chemical Engineering Vrije Universiteit Brussel Brussels Belgium

Mass spectrometry (MS) is an extremely powerful analytical technique, which plays a central role in many scientific fields. The Heaney research group critically reviewed the 2021 applications of ambient ionization mass spectrometry (AIMS), as a follow‐up to the previous year's review paper.^1^ AIMS enables the surface analysis of samples in their native environment in a high‐throughput fashion and is widely used in disease diagnostics, forensics, homeland security applications, and the environmental sciences. Heaney et al. provide an overview of ambient ionization techniques and highlight the applicability in a wide range of scientific fields, either carried out in the laboratory or out in the field. High‐performance liquid chromatography (HPLC) hyphenated to MS is the method of choice to profile oligonucleotide therapeutics. Hannauer et al. summarized the advancements in separation science considering ion‐pairing reversed‐phase chromatography, hydrophilic interaction chromatography, and two‐dimensional liquid chromatography. In particular, the effect of chromatographic elution conditions and column chemistries on retention, resolving power, and MS compatibility are outlined. In addition, recent software developments for the tandem MS analysis of oligonucleotides are discussed. Minkus, Bieber, and Letzel provided a detailed review of the processing of mass‐spectrometric non‐target screening (NTS) data. NTS is an untargeted comprehensive analysis methodology based on high‐resolution MS, where the entire mass range of small organic molecules of anthropogenic origin is recorded within a very short cycling time, leading to complex and large data sets. In particular, Minkus et al. review major contributions that concern the processing of NTS data, prioritization of features, as well as (semi‐) quantitative methods that do not require analytical standards.

Terry et al. provided a detailed overview of applications of surface‐enhanced Raman spectroscopy (SERS) in the environmental sciences. The inherent low intensity provided by Raman signals can be amplified by many orders of magnitude through electromagnetic‐field and chemical/electronic enhancements when the analyte molecule is in close proximity with metal nanostructures. SERS is a promising technique that overcomes inherent limitations of MS, where information of molecular mass is obtained but isomeric differentiation is problematic, and UV‐VIS detection, where contaminants can interfere with the acquired analyte spectra. Terry et al. highlight the characteristics of effective SERS nanosubstrates and methods for the SERS detection of inorganic, organic, and biological contaminants. Moreover, the pros and cons of SERS in environmental detection are discussed and possible avenues for future investigation are provided.

Two reviews were included that focus on the design, development, and applicability of novel stationary phases for LC. Valko described the recent developments of biomimetic stationary phases and chromatographic methods. Biomimetic chromatography may be relatively unknown to most analytical scientists, but the concept is unique as stationary phases containing proteins and phospholipids can mimic the biological environment where drug molecules distribute. Consequently, biomimetic separations allow to assess the in‐vivo interactions of drug molecules and will reduce later stage attrition of candidate molecules due to disadvantageous absorption, distribution, metabolism, and elimination properties. Nechvátalová and Urban reviewed the trends in the development of organic polymer monolithic stationary phases. Macroporous interconnected polymer monolithic stationary phases have been very successfully applied for biomolecule analysis in LC, yielding very high resolving power in gradient‐elution mode. To expand its applicability toward small‐molecule analysis, a lot of effort is directed toward creating new surface chemistries. Nechvátalová describes the latest protocols for controlling the macroporous structure and surface chemistries. In particular, the incorporation of nanoparticles and nanotubes and new developments of the chiral stationary phase are discussed. Also, the development and use of functional monolithic materials for sample preparation are highlighted.

**FIGURE 1:**
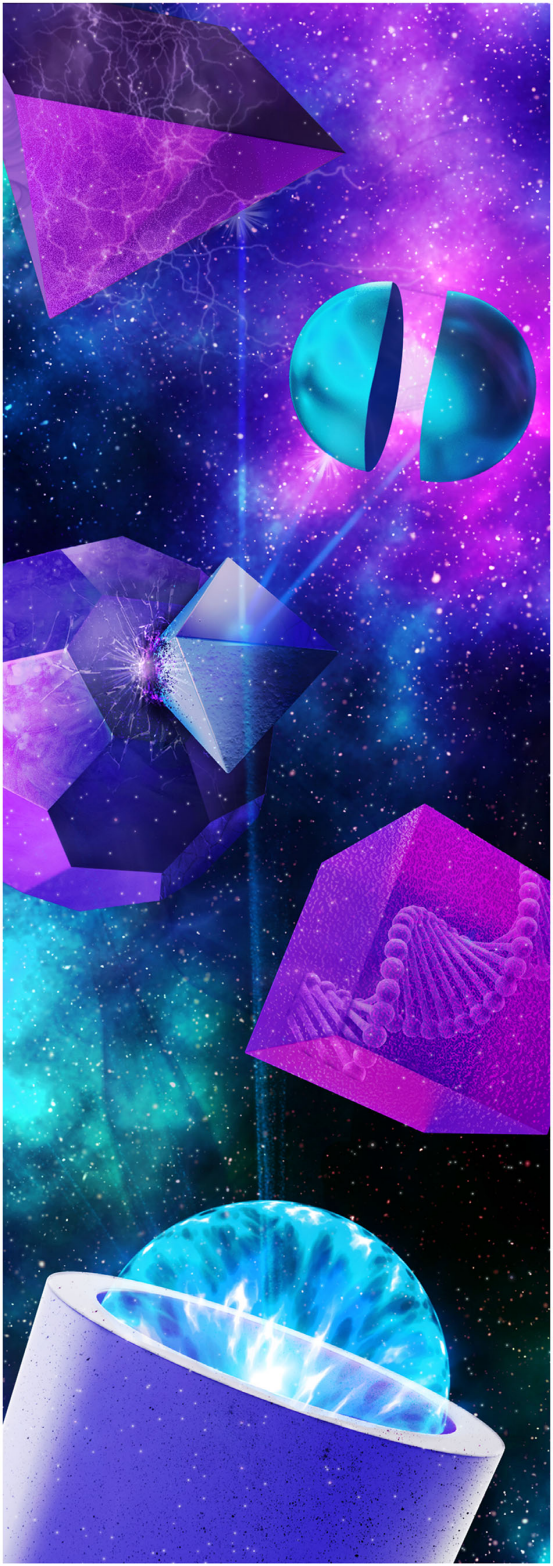
Digital artwork created by Caligrafix Productions that links the different topics presented in this review issue

In 2020, Anal. Sci. Adv. initiated the “Art in Science” initiative which links an artist's impression to a scientific publication.^2^ For this special issue, Calissa Seelen of Caligrafix Productions created a digital artwork that links the different topics described in the review papers in this special issue (Figure 1). I asked Calissa to explain the thought process that led to this illustration, “The greatest scientists are artists as well.” These beautiful words were once used by Einstein and hold a tremendous truth. Science, above else, is a way of creating a better future by generating results that stem from nothing but the researchers’ interesting ideas. Science is an artform and should be valued as one of the greatest gifts we possess as humans. The purpose of science is to comprehend the world around us. Art can be a wonderful translation process of scientific research; showing how scientific results can create a better – and different – future. The two will always be interconnected. This interconnection formed my inspiration for this artist's impression. Many researchers are working on the development of future next‐generation analytical technology.

The image gives a futuristic and artistic look at the progress that has been realized to advance the field of analytical chemistry. The background of this image is set in space because to create a future, researchers must be given space to research what keeps their minds occupied today. Every part of the image has multiple ties with the individual review papers in this special issue. It is the chromatographic column at the bottom, that forms the basis of this artwork, which plays a major role in analytical techniques. The force‐field explosion radiating from this column connects to three‐dimensional objects, representing the important role that irradiation plays in many detection principles. The cube that holds a DNA structure is the only object that reveals its true internal constituents, representative of the search for emerging drug compounds that can affect biochemical pathways. The multifaced shape in this illustration is linked to the search to enhance the structure and chemistry of a monolithic and biomimetic chromatographic support. It is interacting with a crystal‐like structure, which is an important aspect of separation techniques. Reducing the size portrays the importance of many engineering efforts, which is exemplified by a spliced spherical object. The top left corner holds a structure that contains an electrical force field, which is related to mass‐spectrometry interfacing. The objects are situated in the vacuum of intergalactic space, which links to the high‐vacuum condition in mass spectrometry. I hope that we can look forward to a future in which we can create science based on our creative thinking and translate that science through art. In an age where science communication is more important than ever before, I feel very honored to contribute by bringing the scope in a way that is exploring the borders between science and arts.

These review papers in this special issue provide a rapid overview of the latest advances in the technologies highlighted. I was inspired while reading these reviews and expect they will prove to be very valuable for our community, especially for those who are new to these fields. I am very grateful to the authors who contributed and spent their valuable time.
